# Dual Colorimetric Sensor for Hg^2+^/Pb^2+^ and an Efficient Catalyst Based on Silver Nanoparticles Mediating by the Root Extract of *Bistorta amplexicaulis*

**DOI:** 10.3389/fchem.2020.591958

**Published:** 2020-10-22

**Authors:** Farid Ahmed, Humaira Kabir, Hai Xiong

**Affiliations:** ^1^Institute for Advanced Study, Shenzhen University, Shenzhen, China; ^2^College of Physics and Optoelectronic Engineering, Shenzhen University, Shenzhen, China; ^3^Department of Chemistry, Women University of Azad Jammu and Kashmir, Bagh, Pakistan

**Keywords:** colorimetric sensor, green synthesis, silver nanoparticles, catalyst, *Bistorta amplexicaulis*

## Abstract

Environmental pollution derivated from toxic metals and organic toxins is becoming a serious issue worldwide because of their harmful effects on the ecosystem and human health. Here we are reporting an extremely selective and cost-effective colorimetric sensor for simultaneous recognition of Hg^2+^ and Pb^2+^ by using green synthesized silver nanoparticles (AgNPs) mediated from the environmental friendly roots extract of *Bistorta amplexicaulis*. Biogenic synthesized AgNPs were well-characterized by various spectroscopic techniques e.g., UV-vis, FT-IR, XRD, AFM, and Zetasizer. The photophysical potential of synthesized AgNPs toward common metal cations was explored via absorption spectroscopy and colorimetric assay. The hypsochromic shift in the SPR band of AgNPs can easily be detected through naked eyes vision from dark brown to light yellow in the case of Hg^2+^. A substantial reduction in the absorbance of AgNPs was recorded upon mixing with Pb^2+^. AgNPs based colorimetric sensor is highly sensitive toward Hg^2+^ and Pb^2+^ with a limit of detection (LOD) of 8.0 × 10^−7^ M and 2.0 × 10^−7^ M for Hg^2+^ and Pb^2+^, respectively. Furthermore, AgNPs showed promising catalytic activity for the degradation of methyl orange dye. These results demonstrate that *Bistorta amplexicaulis* stabilized silver nanoparticles have potential applications as a colorimetric sensor and an effective catalyst for the degradation of methyl orange.

**Graphical Abstract:**
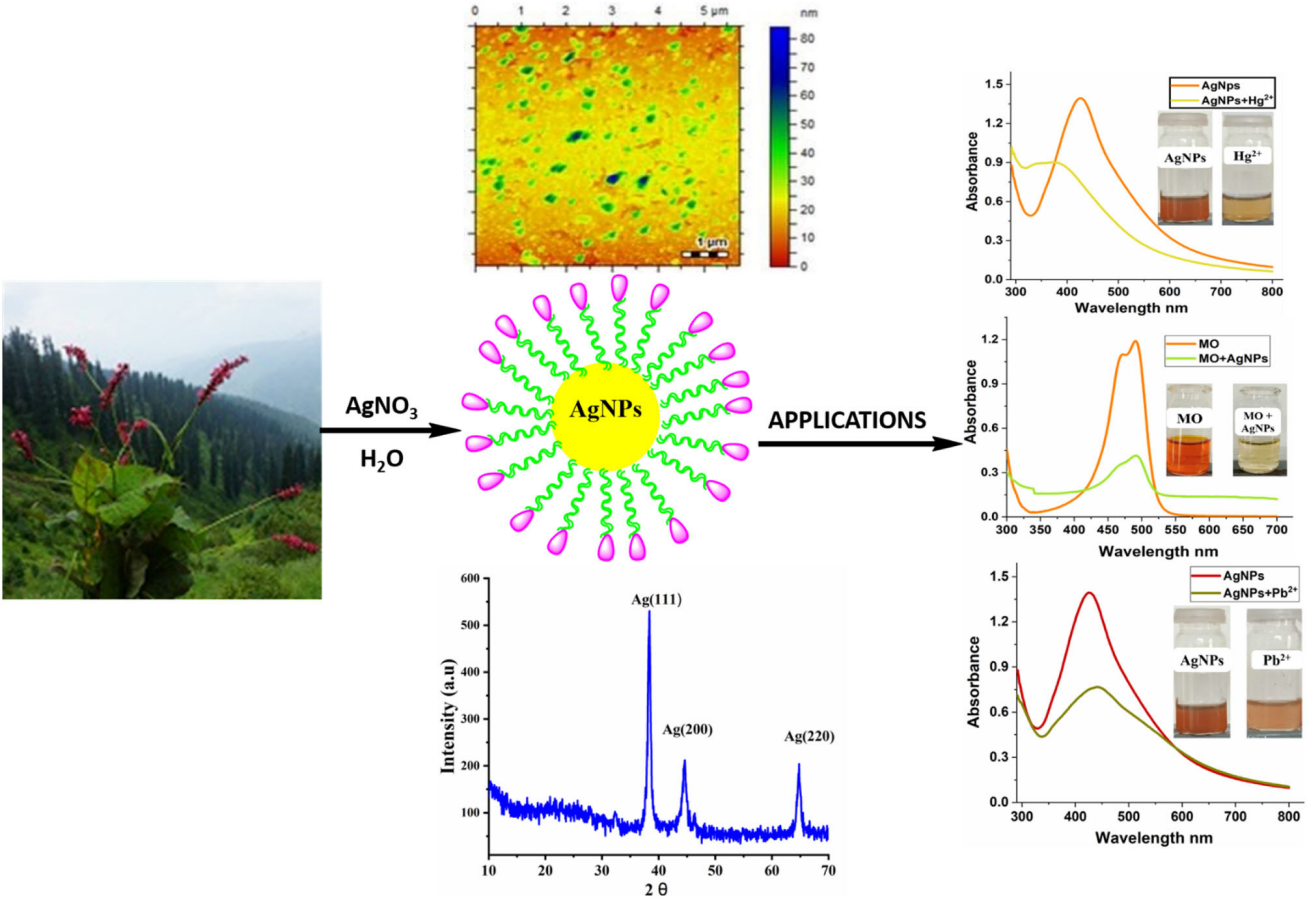
Green synthesized AgNPs were found to be excellent dual colorimetric sensor for Hg^2+^ and Pb^2+^ with a limit of detection of 0.8 and 0.2 μM, respectively. AgNPs as colorimetric sensor offer qualitative and quantitative information by naked-eye visibility without using expensive equipment.

## Highlights

- A highly selective and an inexpensive colorimetric sensor for the detection of Hg^2+^ (1 × 10^−6^ to 1 × 10^−7^ M) and Pb^2+^ (1 × 10^−6^ to 1 × 10^−8^ M) with good linearity was developed by using AgNPs synthesized from *Bistorta amplexicaulis* (root extract).
- AgNPs as colorimetric sensor offer qualitative and quantitative information by naked-eye visibility without using expensive equipment.
- Utilization of plant extract due to its simplicity, non-toxicity, easy availability, relative reproducibility, eco-friendly, low cost, and higher effectiveness. It also does not require intense maintenance of laboratory cultures for nanoparticles synthesis.
- Silver nanoparticles mediating by *Bistorta amplexicaulis* were found to be highly effective catalyst for the degradation of methyl orange dye.

## Introduction

Environmental contamination due to heavy metals and organic dyes is becoming a serious issue worldwide due to their harmful effects on the ecosystem and human health (Duruibe et al., 2007). Heavy metals like cadmium, lead, nickel, chromium, and mercury are significant environmental contaminants in regions with high phylogeny pressure even in trace amounts (Waseem et al., 2014; Paz et al., 2019). Amongst various heavy metal ions, mercury is one of the most toxic metals for human and aquatic life. Elemental mercury and its methyl derivatives are not biodegradable and pose devastating effects on animal and human bodies, which can damage DNA, prevent ligand-receptor binding, damage kidney, liver, disturb the immune system, and even can cause death (Rurack and Resch-Genger, 2002; Ziemba et al., 2006; Hong-Xin et al., 2017). Lead is also considered as extremely toxic metal ions and can cause different health problems in human beings especially in children, senses to epilepsy, unconsciousness, kidney let-down, and even expiry (Papanikolaou et al., 2005; Dooyema et al., 2012). In environment Pb^2+^ in any form cause harms to crops, soil, water, air, and other edible material (Wang et al., 2006). Other organic pollutants also had an adverse effect on human health beside heavy metal ion. About 20% of freshwater pollution has been estimated due to industrial waste containing unconsumed dyes and traces of metal ions. Purification of wastewater using novel environmentally friendly methods is becoming a hot research topic from the last few decades (Yu et al., 2017; Bolisetty et al., 2019).

Several classical methods are reported in the literature for the selective detection of Hg^2+^ and Pb^2+^, including atomic absorption spectrometry, ICP-MS method (Karunasagar et al., 1998; Fong et al., 2007), GC-atomic fluorescence (Nevado et al., 2005), HPLC (Kodamatani et al., 2012), Fluorescence sensor (Chang et al., 2007; Chiang et al., 2008), conjugated polymers (Liu et al., 2007), ratiometric (Yarur et al., 2019), oligonucleotides (Ono and Togashi, 2004; Lin et al., 2011; Lu et al., 2013), proteins (Guo and Irudayaraj, 2011), bioluminescent bacterial sensors (Durand et al., 2015), and electrochemical sensing (Zhu et al., 2017) etc. All the above-mentioned methods used expensive instrumentation, time-consuming sample preparation steps, and more laborious. There is a growing interest for quick on-site analysis with sensors that are capable of distinguishing heavy metal ions on a real-time basis. In this regard, colorimetric sensors have attracted particular consideration for offering qualitative and quantitative information by naked-eye visibility without using sophisticated equipment (Tang and Li, 2017; Choudhary et al., 2020; Kateshiya et al., 2020). Noble metal nanoparticles (AuNPs, AgNPs) are extensively used in the field of sensing and biosensing due to their exceptional optical properties. Silver nanoparticles (AgNPs) have gained remarkable attention in the area of colorimetric sensors because of their distinctive properties (Farhadi et al., 2012; Wang et al., 2012; Jarujamrus et al., 2015; Singh R.K. et al., 2018; ul Ain et al., 2019). Silver nanoparticle exhibits a high excitation coefficient and specific optical properties in the visible region. Conventionally nanoparticles were synthesized by physical and chemical methods. The chemical synthesis of AgNPs contributes a number of the disadvantages like use to reducing agents, harmful chemical adsorbs on the external surface of NPs having damaging effects in the pharmaceutical applications. Alternatively, green synthesis of NPs using root and leaf exact of plants has gained much interest due to an environment-friendly approach (Dhand et al., 2016; Jebril and Dridi, 2020). The utilization of root and leaf extract of plants has shown to be better methods because of slower kinetics, economical, eco-friendly, and fewer biohazards (Wang et al., 2014; Veisi et al., 2018). The phytochemicals present in plant extracts such as protein, amino acid, polyphenols, carbohydrates *etc*. may act as reducer and stabilization agents in the synthesis of biogenic silver nanoparticles (Gomathi et al., 2020). The presence of diverse functional groups in the capping facilitates the binding of metal ion on to the surface of silver nanoparticles. A different part of plant extracts (fruit, flower, leaves, and root) can be used to reduce and stabilize the metal/metal oxide nanoparticles in the “one-pot” synthesis method. Due to the tunable size and distance-dependent optical properties of metallic nanoparticles, they have been preferably used for the recognition of heavy metal ions in contaminated water systems (Annadhasan et al., 2014). Many researchers have used a green synthesis approach for the synthesis of metal/metal oxide nanoparticles *via* plant leaf extracts to further explore their various applications (Koduru et al., 2018). The advantages of using metal NPs as colorimetric sensors for heavy metal ions in environmental systems/samples include simplicity, cost-effectiveness, and high sensitivity at sub-ppm levels. Plant extracts stabilized AgNPs and AuNPs are used as colorimetric probe for the recognition of heavy metal ions (cadmium, mercury, lead, chromium etc.) in water (Singh J. et al., 2018).

Here we are reporting the biogenic synthesis of AgNPs using *Bistorta amplexicaulis* root extract for the colorimetric recognition of Hg^2+^ and Pb^2+^ ion for the first time. Biogenic AgNPs were characterized by using UV-visible, FTIR, XRD, AFM, and Zetasizer analysis. The photophysical potential of NPs was studied using UV-visible spectroscopy and colorimetric assay. Newly synthesized AgNPs were found to be a highly selective colorimetric sensor for Hg^2+^ and Pb^2+^ even in the presence of several competitive metal ions. The catalytic activity of nanoparticle was also evaluated which revealed that AgNPs could be used as a catalyst for the degradation of methyl orange.

## Experimental Section

### Martials and Methods

Roots of *Bistorta amplexicaulis* plant was collected from Ghangachoti Bagh Azad Kashmir. Chemicals including AgNO_3_, NaNO_3_, KCl, CaCl_2_, SnCl_2_, BaCl_2_·2H_2_O, HgCl_2_, PbCl_2_, CuSO_4_, Mg(NO_3_)_2_, Ni(NO_3_)_2_·6H_2_O, AlCl_3_, and CuSO_4_·5H_2_O were purchased from chemical companies i.e., Merck or Sigma Aldrich and used without any further pretreatment. A calculated amount of AgNO_3_ and each metal salt were added in distilled water to prepare a stock solution.

### Preparation of Plant Extract

Plants' roots were carefully washed three times with deionized water to remove the dust particles and dried in shadow then grinded to a fine powder. After that 5 g roots powder of *Bistorta amplexicaulis* was socked in 100 mL of H_2_O and stirred for 1 h at 25°C. Subsequently, the mixture was filtered via normal filter paper, and obtained root extract solution was used for nanoparticle synthesis.

### Biogenic Preparation of AgNPs

Nanoparticles were synthesized using a green chemistry approach without using any toxic reducing or capping agents. For the preparation of Bistorta *amplexicaulis* stabilized AgNPs plant extract solution and AgNO_3_ (1 mM) solution was mixed in various ratios (1:1, 1:5, and 1:10 v/v), and resulted mixture was stirred at 25°C for 30 min. The formations of nanoparticles were examined by the color change of the solution. Different parameters related to the synthesis and stability of nanoparticles was also optimized.

### Characterization of Nanoparticles

UV-visible spectrophotometer-1800 (Shimadzu, Japan) was used for the characterization and sensing application of AgNPs. The shape and average size of AgNPs were dogged through Agilent 5500 atomic force microscope (AFM). Crystalline nature of synthesized NPs was studied by XRD analysis using X-ray diffractometer (Shimadzu, XRD-6000). FTIR spectrum was recorded utilizing KBr discs method by using FTIR Bruker-EQUINOX-55 at encompassing conditions.

### General Procedures for Sensing Experiments

The sensing potential of the synthesized nanoparticles toward metal cations was explored using UV-visible spectroscopy. Generally, 1 mL solution of nanoparticles was mixed with 1 mL solution of different metal ions (100 μM) at 25°C. Colorimetric response and alteration in absorption intensity were examined using UV-visible spectroscopy. A detailed procedure for different sensing experiment is provided in the Supplementary Information.

### Tap Water Experiments

Tap water samples were collected from the chemistry lab of Women University Bagh Azad Kashmir Pakistan and were used without filtration. Tap water samples were spiked with a known concentration of Hg^2+^ (100 μM) and Pb^2+^ (100 μM). Nanoparticles solution was mixed with Hg^2+^ and Pb^2+^ solution (prepared in tap water) under similar experimental conditions and the changes in the absorption spectrum were recorded.

### General Procedures for the Degradation of Methyl Orange Dye

For degradation experiments, 5 mL of methyl orange solution (0.1 mM), a fixed amount of silver nanoparticles (1 mg) were mixed to stir at room temperature. The degradation of methyl orange was checked by time-based UV-visible spectra. The sample was stirred for maintaining the equilibrium of the working solution and recorded UV- spectra before and after stirring. Before stirring at “0” time, the first reading was taken and then placed the sample for stirring to observe the color change at different times. Then UV- visible spectra of these samples were recorded and calculate a standard error of each reading.

## Results and Discussion

### Characterization of AgNPs

**Figure 1:**
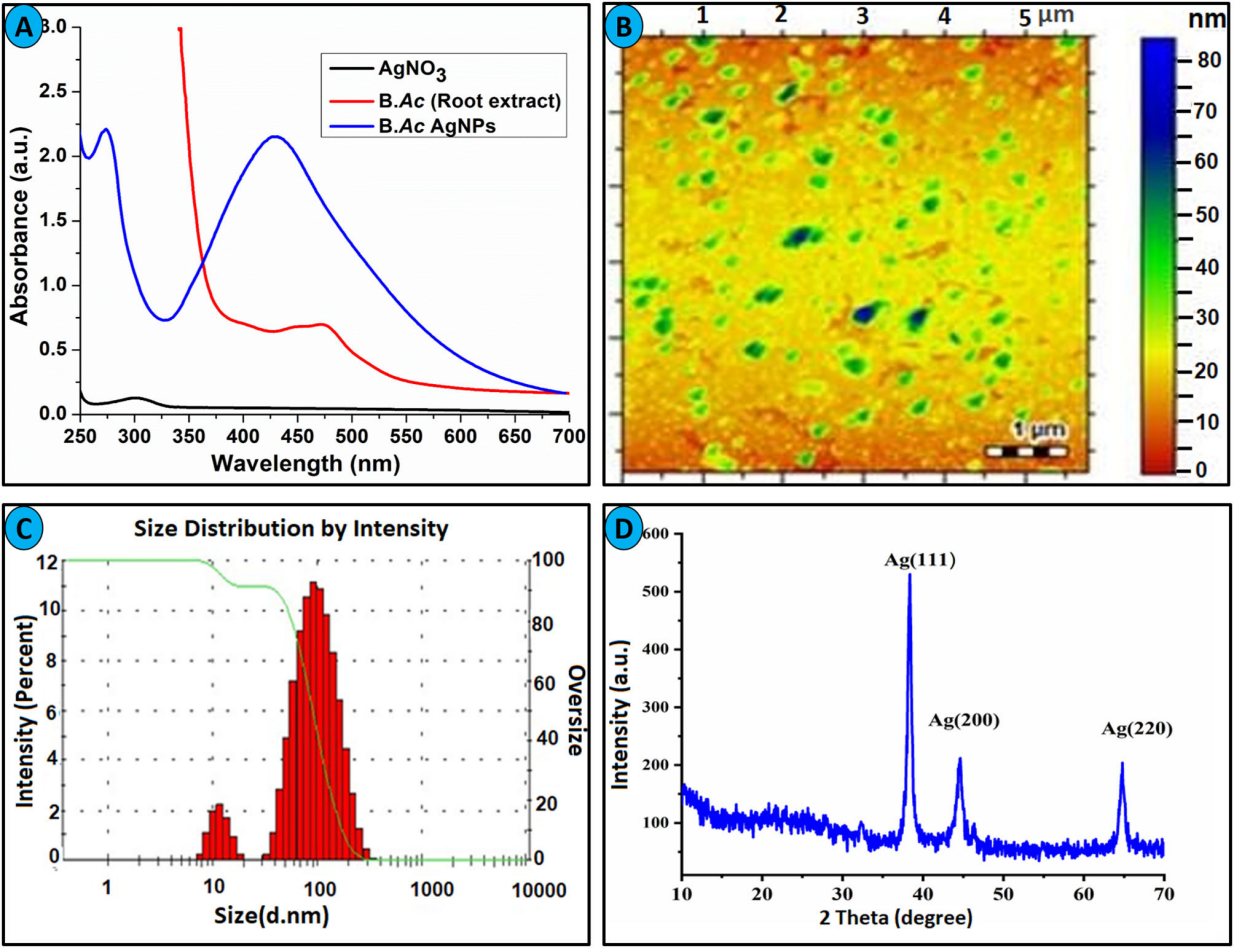
**(A)** UV-visible spectra of *Bistorta amplexicaulis* root extract stabilized AgNPs. **(B)** AFM image of AgNPs. **(C)** Average size distribution by intensity. **(D)** XRD spectrum of AgNPs.

*Bistorta amplexicaulis* stabilized AgNPs were characterized *via* various spectroscopic techniques including UV-visible, XRD, AFM, Zetasizer, and FTIR. Silver nanoparticles contained distinctive optical properties in which they interact strongly with specific wavelengths of light. Electrons move freely in AgNPs because the difference between valence and conduction band is minimum. Due to these free electrons, surface plasmon resonance (SPR) absorption band is formed. The SPR band formation is because of the collective oscillation of AgNPs and electrons are in resonance with light waves (Zhang et al., 2016). *Bistorta amplexicaulis* stabilized silver NPs showed characteristic nanosized particles band at 427 nm as shown in Figure 1A. Carbohydrates, amino acid, and polyphenolic compounds present in the aqueous extracts of *Bistorta amplexicaulis* perform a dual role as reductant along with capping agents in the stabilization of AgNPs (Supplementary Table 1). Phenolic hydroxyl groups present in plant extract are well-known reductants for Ag^+^ in the synthesis of polydispersed AgNPs. Silver nanoparticle showed maximum absorption intensity at 5:1 of (AgNO_3_*: Bistorta amplexicaulis* v/v), while upon the increasing concentration of plant extract solution did not produce any significant change in the wavelength of nanoparticles while absorption intensity decreased slightly. The particle size of newly synthesized nanoparticles was determined using AFM and zeta sizer analysis. AFM investigation was employed to observe the surface morphology and an average particle size of the synthesized nanoparticle. AFM analysis showed that most of AgNPs are spherical with an average size between 50 and 60 nm (Figure 1B). The bigger particles in the AFM image can be described as the assembly of smaller NPs. The size of nanoparticles was also measured by Zetasizer analysis which demonstrated that nanoparticles have an average size of around 60 nm with a poly dispersive index value of 0.39 (Figure 1C).

*Bistorta amplexicaulis* stabilized nanoparticles were also characterized by XRD analysis to confirm the crystal nature of synthesized nanoparticles. The XRD pattern exhibited distinctive Bragg reflections, which may be cataloged on the basis of the specific crystalline structure of silver nanoparticles. The XRD graph showed three obvious peaks for AgNPs at 38.2° (111), 44.3° (200), and 64.5° (220) as depicted in Figure 1D. These Bragg reflections resembled the crystalline planes of the face-centered cubic (fcc) crystal lattice of metallic silver. Thus, XRD analysis confirms the formation of AgNPs in the shape of the nanocrystal. The obtained XRD pattern of AgNPs is well-matched with the XRD results of plant extract stabilized nanoparticles reported in the literature (Umamaheswari et al., 2018; Santhosh et al., 2019). FT-IR spectroscopy was employed to find out the nature of the functional group taking part in the stabilization of AgNPs. By matching the FT-IR spectrum of *Bistorta amplexicaulis* with AgNPs, biomolecules of different classes present in the root extract of *Bistorta amplexicaulis*, and their conjugation with silver is confirmed from 4,000 to 400 cm^−1^ range (Supplementary Figure 3). The FT-IR spectrum of the plant extract showed characteristic peaks of for -OH, -CH, -C=O, and -NH functional groups at 3,403, 2,950, 1,737, and 1,622 cm^−1^, correspondingly. All the peaks presents indicate the presence of polyphenolic, carbohydrates, and protein-based function constituents in the plant extract. The sharp hydroxyl and N-H(bend) peak at 3,403 and 1,622 cm^−1^ are disappeared in the FT-IR spectrum of AgNPs (Supplementary Figure 3) while the intensity of the carbonyl group(-C=O) decreased significantly. These changes in the FT-IR spectrum of nanoparticles endorsed the major involvement of -OH and -NH containing biomolecules (present in root extract) in the stabilization of nanoparticles.

### Stability of *Bistorta amplexicaulis* Coated AgNPs

**Figure 2:**
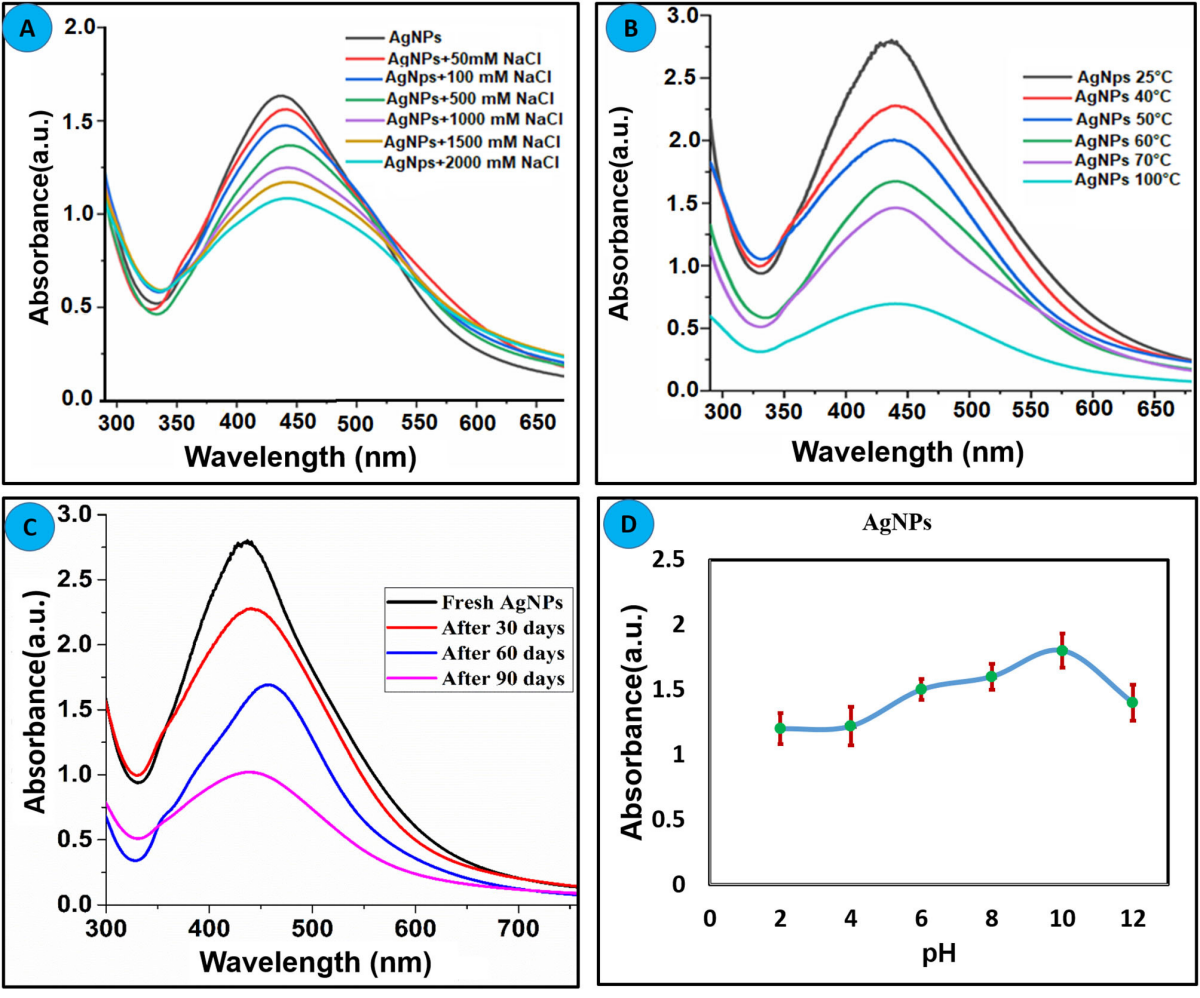
Effect of the different parameters on the stability of green synthesized AgNPs **(A)** Effect of electrolyte concentration. **(B)** Thermal stability of AgNPs. **(C)** Effect of time on the stability of AgNPs. **(D)** Effect pH on the stability of AgNPs.

Nanoparticles stability under different circumstances such as pH, heat, and electrolyte concentration is considered as an important characteristic for the real application. To define the stability of synthesized silver nanoparticles, different experiments were carried out and results are summarized in Figure 2. Different concentration of NaCl (0.05–2 M) was mixed with AgNPs, and alteration in the absorbance was recorded as displayed in Figure 2A. It can be concluded that AgNPs are highly stable up to 1 M NaCl concentration. At a high concentration of NaCl (2 M), the broadness in the absorption band and decline in the absorbance of nanoparticles is attributed to the aggregation effect of the chlorine ion (Bae et al., 2002). To explore the thermal stability of AgNPs, experiments were conducted at an elevated temperature. The nanoparticle solution was heated to the different temperatures, and change in absorption intensity was recorded as shown in Figure 2B. *Bistorta amplexicaulis* coated AgNPs were stable up to 70°C whereas a significant decrease in the absorption intensity was observed at a higher temperature without any precipitation which attributed to the dominant electronic dephasing mechanism (Link et al., 1999). To estimate the effect of time on the stability of nanoparticles, change in the absorption intensity was recorded in a different time interval as depicted in Figure 2C. It can be concluded from the figure that nanoparticles are quite stable up to 60 days but after that stability decreased. The pH of the medium plays a critical role in the synthesis and stabilization of the biogenic synthesized nanoparticles. The main effect of the reaction pH is its stability to alter the electrical charges of biomolecules which might affect their capping and consequently the growth of the nanoparticles (Priya et al., 2016). The effect of pH on the stability of nanoparticles pH-dependent was performed as depicted in Figure 2D, and it can be concluded that synthesized nanoparticles are stable over a wide range of pH (2–12). A slight decrease in the absorption intensity of nanoparticles was observed in high pH value which might be due to deprotonation of capping agents.

### Sensing Application of AgNPs Toward Metal Ions

**Figure 3:**
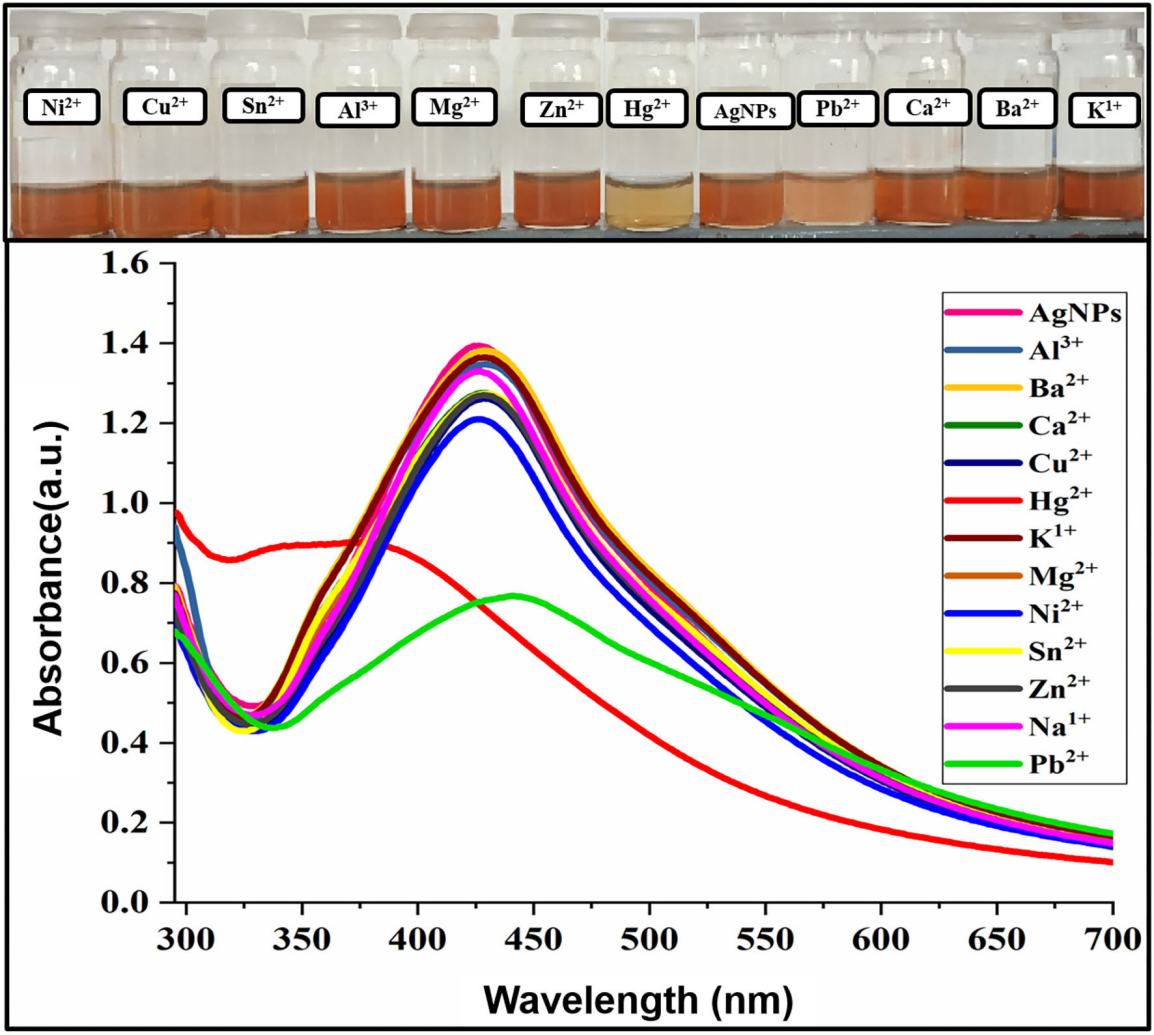
Change in absorption intensity and the colorimetric response of AgNPs upon the addition of several metal ions.

The photophysical potential of newly synthesized nanoparticle toward metal cations was studied via colorimetry and absorption spectroscopy. For the recognition study 1.0 mL of nanoparticles was mixed with 1.0 mL of various metals (100 μM) at room temperature and changes in absorption intensity were recorded. The addition of Hg^2+^ and Pb^2+^ in AgNPs solution gives a colorimetric response within 5 min as the dark brown color of AgNPs change to light yellow upon the addition of Hg^2+^ and light brown in the case of Pb^2+^ revealing the binding of Hg^2+^ and Pb^2+^ with AgNPs as depicted in Figure 3. The absorption spectra showed the broadness and blue shift in the absorption band upon interaction with mercury Hg^2+^ and hypochromic shift in presence of lead Pb^2+^. All other metals, including Cu^2+^, Zn^2+^, Mg^2+^, Ni^2+^, Na^+^, K^+^, Al^3+^, Ba^2+^, Ca^2+^, and Sn^2+^ did not make any alteration in the color and absorption spectrum of the nanoparticles. Hg^2+^ and Pb^2+^ were the only two metals that caused the remarkable change which can be distinguished by the naked eye and absorption band of AgNPs. These results demonstrated good selectivity for these metal ions over a wide range of other tested metals, thus AgNPs have specific binding sites for mercury (Hg^2+^) and lead (Pb^2+^). The presence of organic functional groups (-COOH, -NH_2_, -OH, etc.) in the stabilizing agent facilitates the binding of mercury on to the surface of AgNPs (Farhadi et al., 2012; Kamali et al., 2016).

**Scheme 1:**
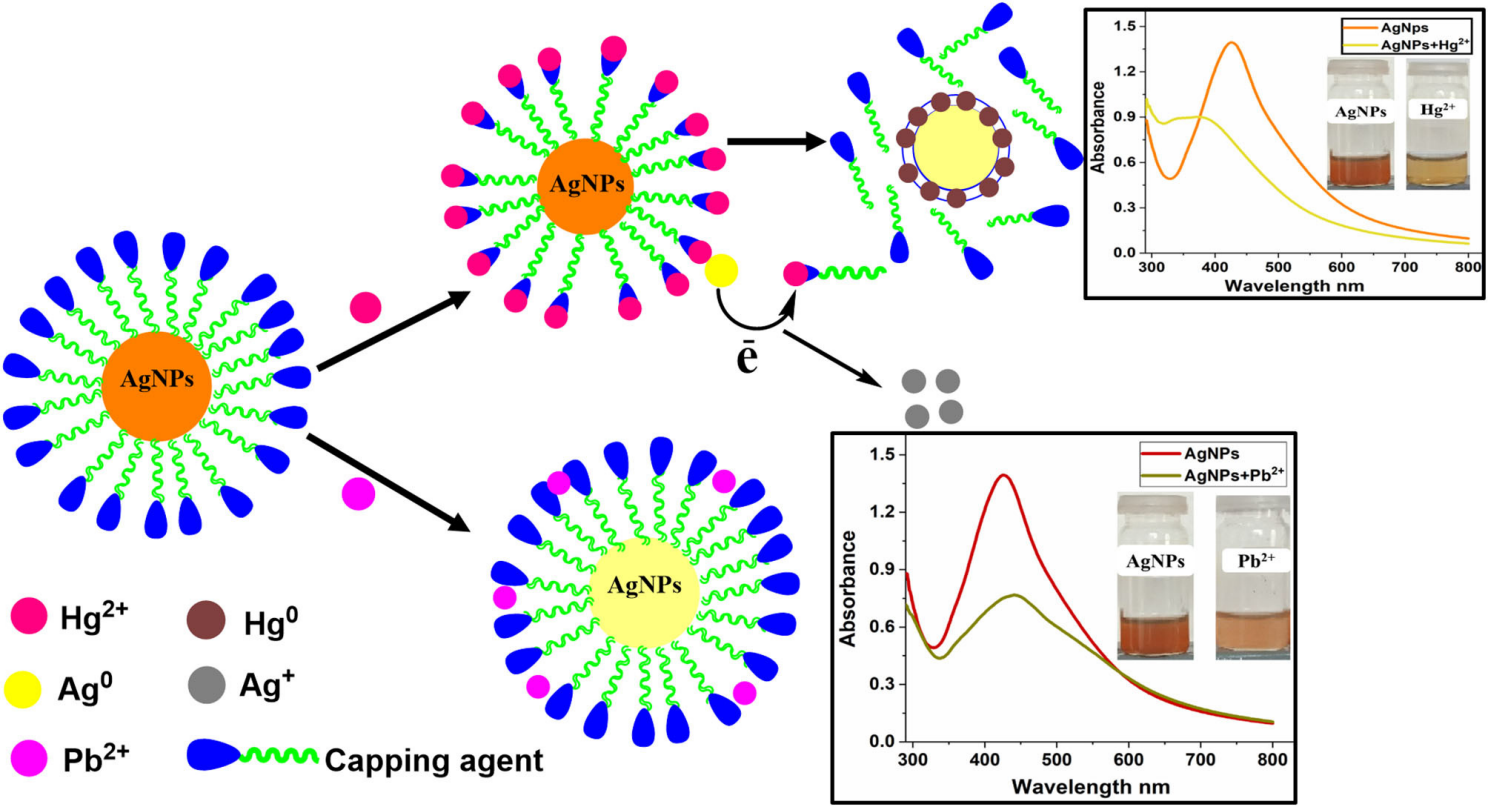
Possible sensing mechanism for Hg^2+^and Pb^2+^ using *Bistorta amplexicaulis* stabilized AgNPs.

The possible mechanism for the selective recognition of Hg^2+^ can be explained based on redox reaction taking place at the surface of the AgNPs, due to the differences in the standard potential of 0.8 V (Ag^+^/Ag) and 0.85 V (Hg^2+/^Hg). Upon addition of Hg^+2^ to the colloidal AgNPs suspension, the presence of organic capping agents on the surface of AgNPs stimulates electrostatic–ionic attractions between the NPS and Hg^2+^ (Scheme 1). The added mercury reacts with the Ag^0^ core by redox reaction and displaces the capping agents present on the surface of the AgNPs. Flavonoids, carbohydrates, amino acids, and their derivatives are present in plant extracts and are known to form complexes with heavy metal cations (Sumi et al., 2017). As a result of these redox reactions taking place between Ag^0^ and mercury, Hg^2+^ gets reduced to Hg^0^ on the nanoparticle surface and Ag^0^ oxidized to Ag^+^. As the AgNPs are converted to Ag^+^ ions by added mercury, the colloidal solution becomes colorless. The result demonstrates that the accessible Ag^0^ in the colloidal suspension has been completely oxidized by the added quantity of mercury.

#### Effect of Mercury (Hg^2+^) and Lead (Pb^2+^) Concentration

**Figure 4:**
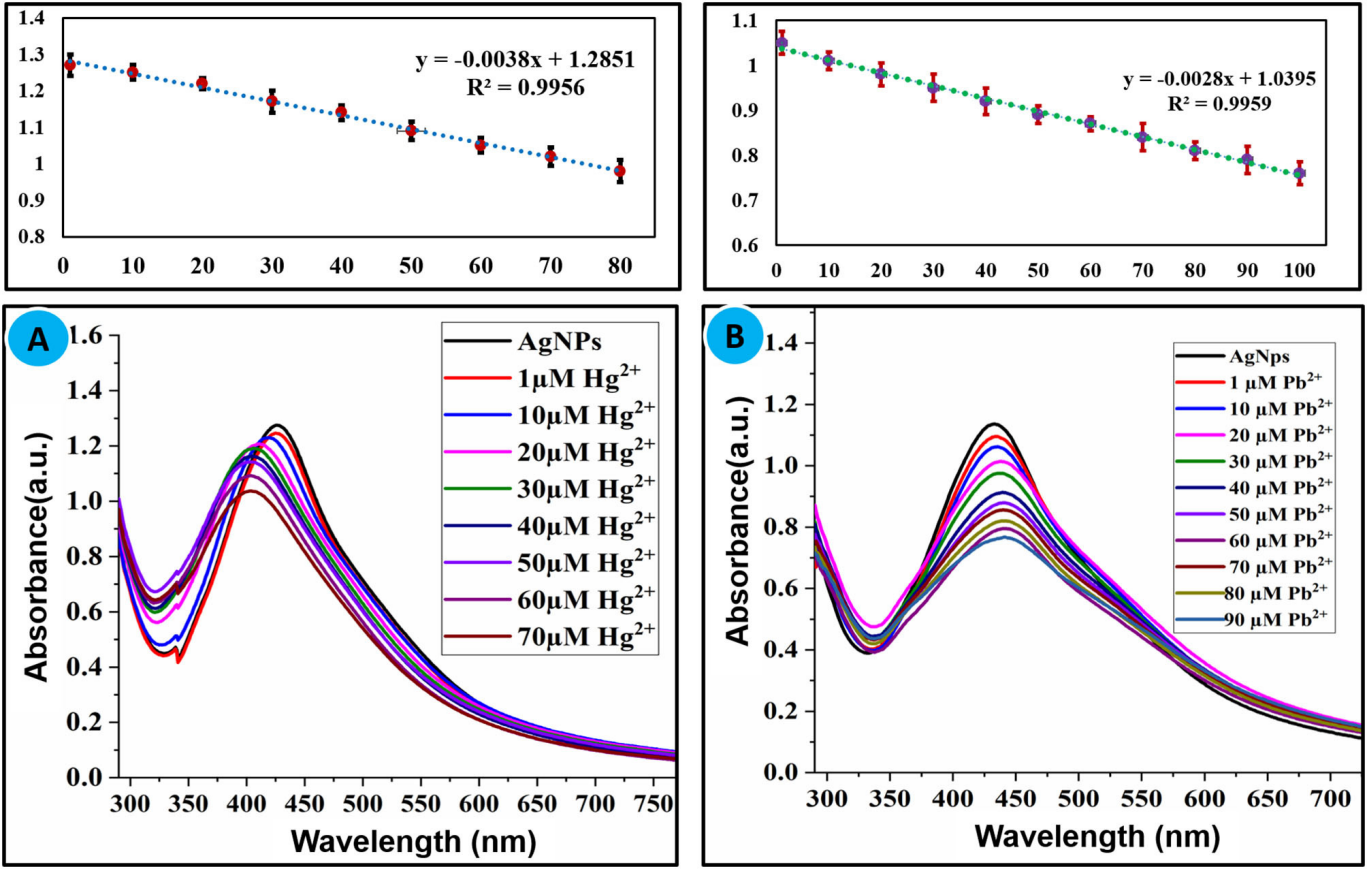
**(A)** Effect of concentration of Hg^2+^ (1–70 μM) and **(B)** Pb^2+^ (1–90 μM) on the absorption intensity of AgNPs.

Sensitivity toward a particular analyte is a vital parameter of a sensory system. Maintaining the concentration of nanoparticles constant, the concentration of Hg^2+^(1–70 μM) and Pb^2+^(1–90 μM) was varied and the effect of different concentrations of selected ions on the absorption intensity of AgNPs was recorded as shown in Figures 4A,B. A linear relationship between concentration and absorption intensity was found for both Hg^2+^ and Pb^2+^ with a correlation factor of *R*^2^ = 0.9956, *R*^2^ = 0.9959 for the range of 1–70 μM, and 1–90 μM, respectively. The limit of detection of Hg^2+^ and Pb^2+^ was calculated using a slope of the straight-line equation and standard deviation of the blank using the following formula LOD = F × SD/b. The lower limit of detection for Hg^2+^ and Pb^2+^ was calculated to be 8.0 × 10^−7^ M and 2.0 × 10^−7^ M correspondingly. The comparison of the present study with reported methods for the recognition of Hg^2+^ and Pb^2+^ is shown in Tables 1, 2, respectively. As can be seen from the tables that are lower limit of detection for Hg^2+^ and Pb^2+^ is comparable with recently reported methods. The comparison shows that biogenic synthesized nanoparticle is a very efficient, cost-effective, and more importantly environmental-friendly sensor for the detection and quantification of the micromolar concentration of Hg^2+^ and Pb^2+^ ions.

**Table 1:** Comparison of current study with reported methods for the detection of Hg^2+^.

**Responsive technique**	**Sensor**	**Limit of detection (M)**	**Synthetic approach**	**References**
Colorimetry	Melamine stabilized AgNPs	2 × 10^−6^	Chemical method	Kailasa et al., 2018
Colorimetry	PEG-luteolin stabilized AgNPs	2 × 10^−6^	Chemical method	Qing et al., 2018
Colorimetric and UV-visible spectroscopy	Phenylenediamine functionalized silver nanoparticles (AgNPs)	8.0 × 10^−7^	Chemical method	Bothra et al., 2013
Cyclic voltammetry	*Arbutus andrachne* Stabilized AgNPs	8.43 × 10^−6^	Green chemistry	Eksin et al., 2019
Colorimetric and UV-visible spectroscopy	*Syzygium aqueum* extract stabilized AgNPs	8.7 × 10^−7^	Green chemistry	Firdaus et al., 2017
Colorimetric and spectrophotometric	*Bistorta amplexicaulis* stabilized AgNPs	8.0 × 10^−7^	Green chemistry	Current study

**Table 2:** Comparison of the current study with reported methods for detection of Pb^2+^.

**Responsive technique**	**Sensor**	**Limit of detection (M)**	**Synthetic approach**	**References**
UV-Visible and Colorimetry	Gold nanoparticles	5.0 × 10^−7^	Chemical method	Wei et al., 2008
UV-Visible and Colorimetry	Schiff base sensor	5.4 × 10^−6^	Chemical method	Wang et al., 2018
UV-visible and fluorescence	Quinoline–coumarin based sensor	5.0 × 10^−7^	Chemical method	Meng et al., 2018
Voltammetry	Bismuth oxycarbide/nafion electrode	3.5 × 10^−6^	Chemical method	Zhang et al., 2020
Colorimetric and UV-visible spectroscopy	*Bistorta amplexicaulis* stabilized AgNPs	2.0 × 10^−7^	Green approach	Current study

#### Interfering Study

**Figure 5:**
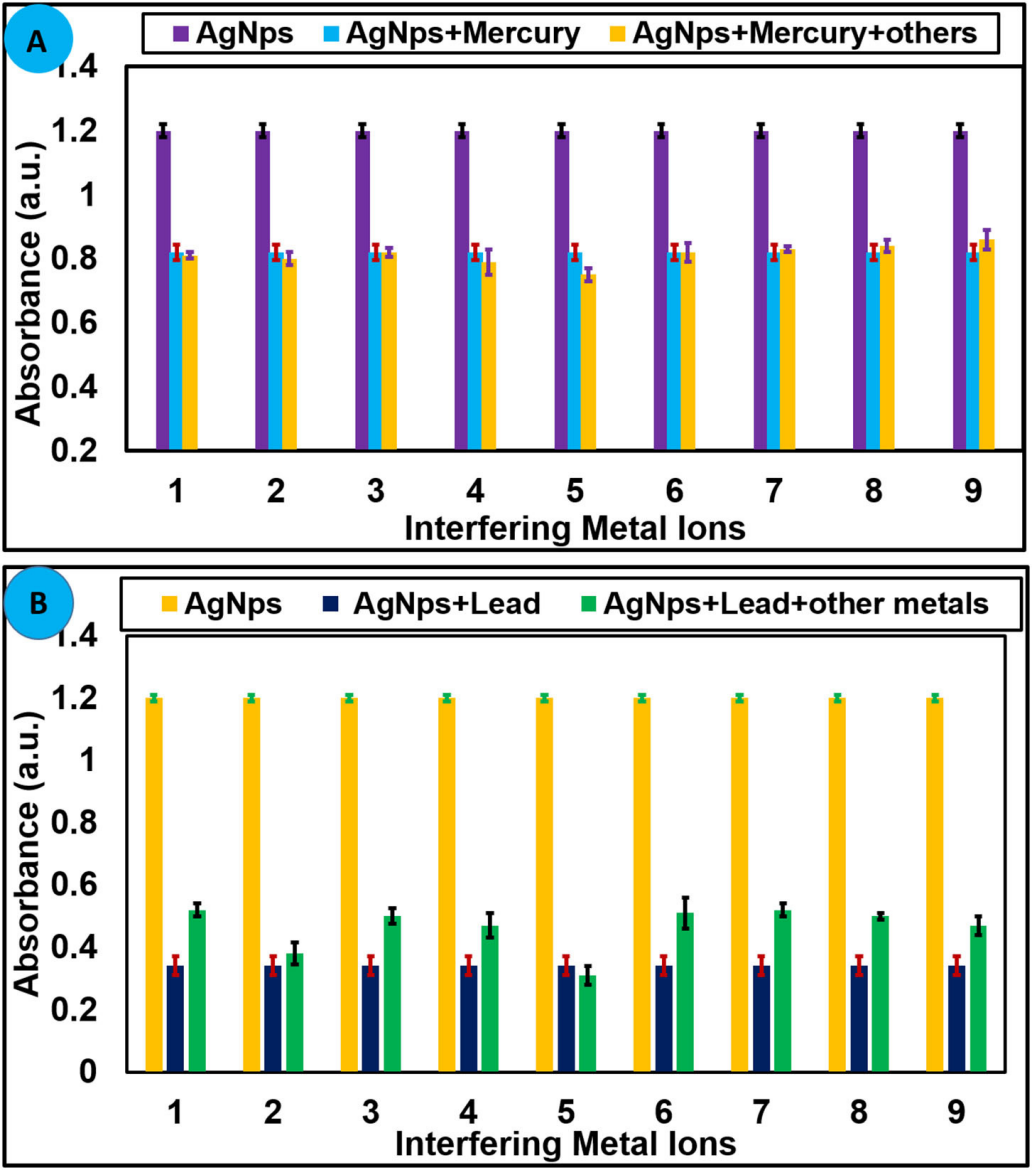
**(A)** Selectivity of AgNPs toward Hg^2+^ (70 μM) at 427 nm and **(B)** toward Pb^2+^ (100 μM) in presence of several metal cations. 1 = Ni^2+^, 2 = Zn^2+^, 3 = Mg^2+^, 4 = Ca^2+^, 5 = Sn^2+^, 6 = Cr^3+^, 7 = K^+^, 8 = Na^+^, 9 = Al^3+^, and 10 = Cu^2+^.

Detection of the targeted analyte with high selectivity in the presence of other similar analytes is an essential property of a sensor when used for analytical applications. To find out the selectivity of AgNPs toward selected metal ions, competitive experiments were performed in the existence of other metal ions. Several metal ions (100 μM) were treated with AgNPs in the presence of Hg^2+^ (100 μM) and Pb^2+^ (100 μM) under similar experimental conditions, and variation in the absorbance was recorded. It can be concluded from competitive experiments that no substantial change in the absorbance was observed with the addition of interfering metals ions, and the absorption intensity was nearly parallel to that caused by Hg^2+^ and Pb^2+^ alone as shown in Figures 5A,B. This undoubtedly suggests that AgNPs are highly selective in the sensing of Hg^2+^ and Pb^2+^without any interference from other tested metal ions.

#### Effect of pH and Binding Stoichiometry

**Figure 6:**
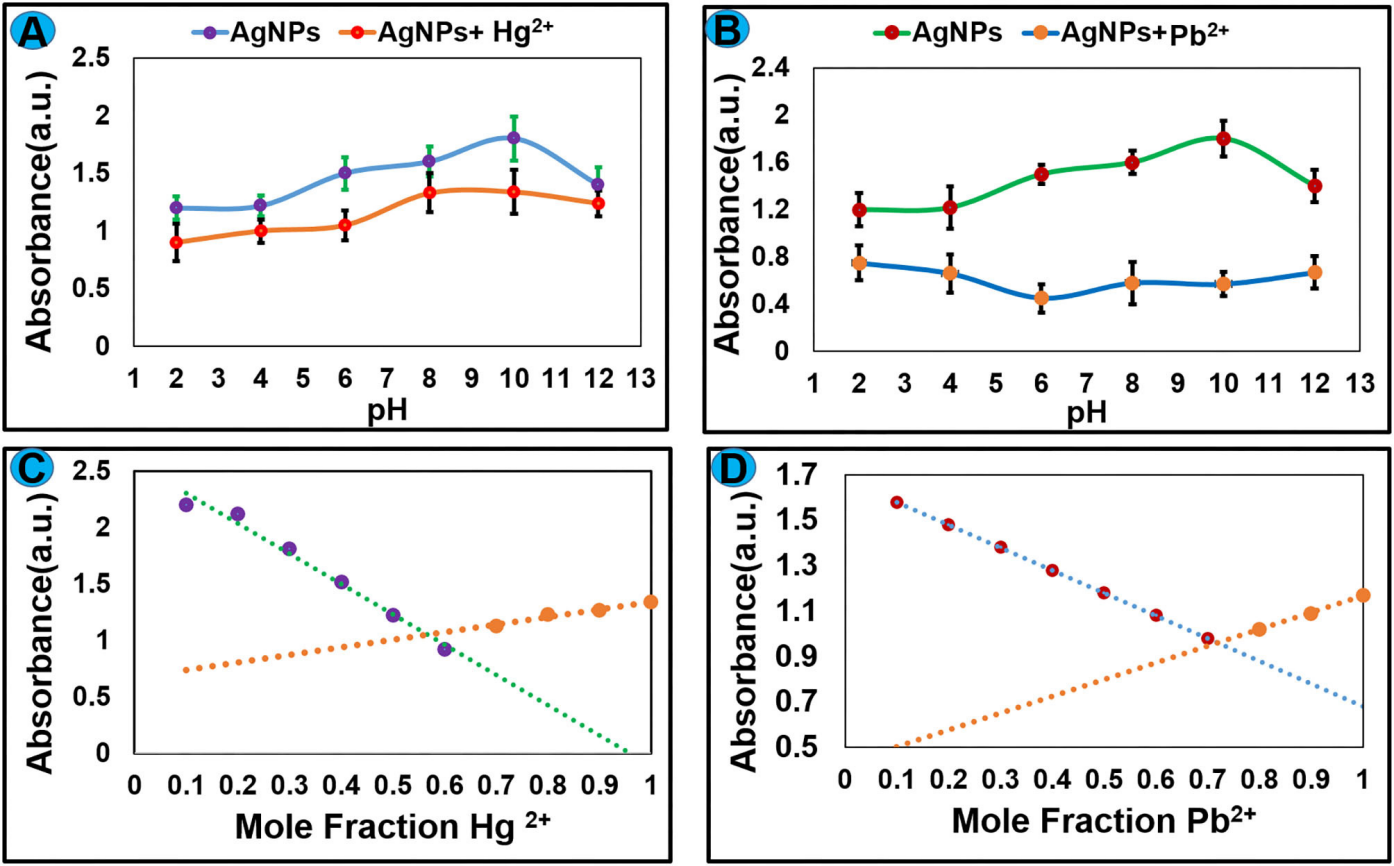
The effect of pH on the complex formation between AgNPs and **(A)** Hg^2+^ and **(B)** Pb^2+^. Job's plot for binding stoichiometry between AgNPs and selected metal ions **(C)** Hg^2+^ and **(D)** Pb^2+^.

The pH value of the medium had a remarkable effect on the stability and sensing applications of nanoparticles. To optimize the effect of pH on the sensing potential of AgNPs toward Hg^2+^ and Pb^2+^, experiments were carried out under different pH from 2 to 12. An aqueous solution of NaOH (0.1 M) and HCl (0.1 M) was used to control the pH value. Absorption spectra were recorded at room temperature and the graph was plotted between absorbance vs. pH as shown in Figures 6A,B. The result revealed that AgNPs can be employed for the recognition of Hg^2+^ and Pb^2+^ over a wide range of pH. The binding stoichiometry of the AgNPs-mercury complex and AgNPs-lead complex was monitored by the job's plot method. Keeping the total concentration constant mole fraction value of AgNPs and selected metal ions were varied from 0.1 to 0.9. Change in the absorbance was recorded, and the graph was plotted between absorbance and mole fraction of Hg^2+^ and Pb^2+^ ions. It can be seen from Figures 6C,D that maximum reduction in the absorption intensity was observed at a mole fraction value of around 0.6. These results suggested 1:1 binding stoichiometry between AgNPs and selected metal ions.

#### Detection of Hg^2+^ and Pb^2+^ in Tap Water

**Figure 7:**
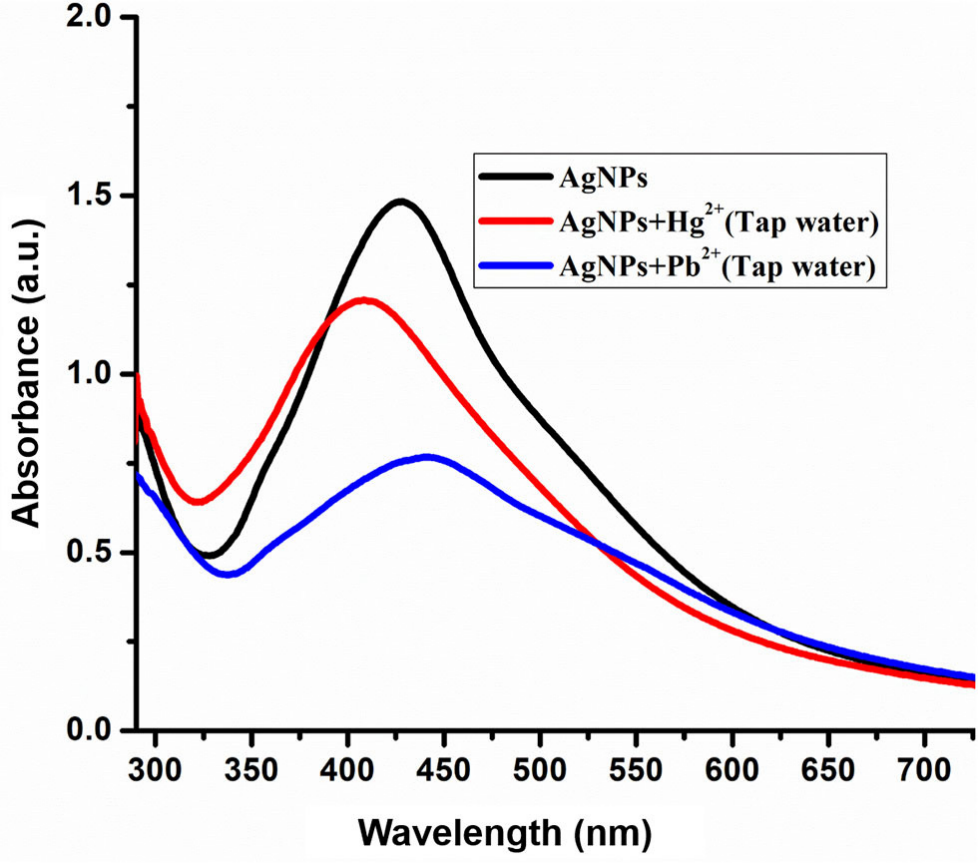
Spectra shows the detection of Hg^2+^ (100 μM) and Pb^2+^ (100 μM) in laboratory tap water.

The practical applicability of AgNPs detection of selected metal ions was carried out in the tap water sample. Tap water samples were collected from Bagh Azad Kashmir and spiked with a known concentration (100 μM) of Hg^2+^ and Pb^2+^ ions. The freshly prepared solution of Pb^2+^ and Hg^2+^ were mixed with AgNPs solution at room temperature and change in absorbance was noted as presented in Figure 7. Tap water analysis revealed that a component present in tap water didn't interfere in the detection of Hg^2+^ and Pb^2+^. Hence green synthesized AgNPs can be employed for the selective detection of Hg^2+^ and Pb^2+^ ion in the environmental samples without any interference from other similar metal ions.

### Evaluation of the Catalytic Activity of AgNPs

**Figure 8:**
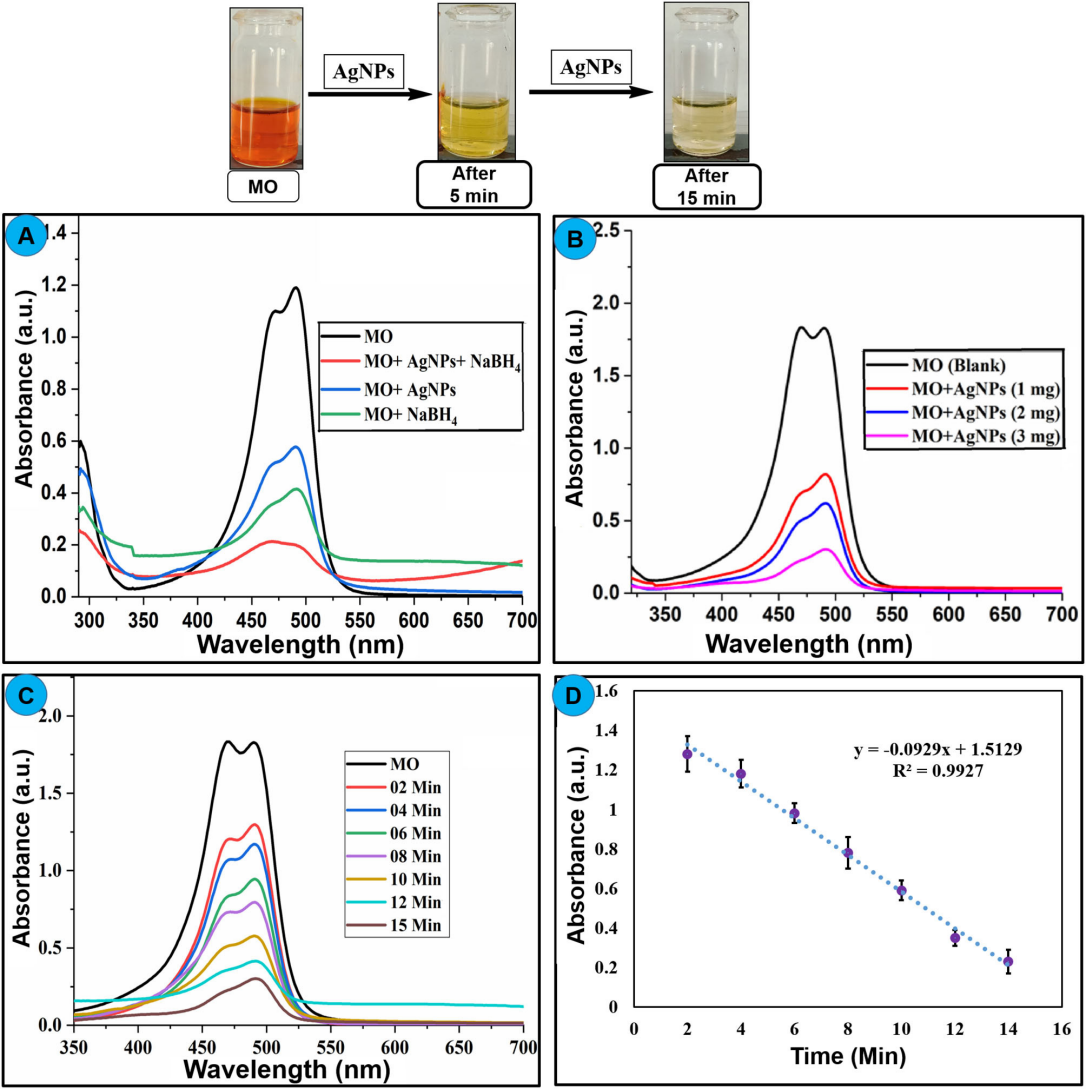
**(A)** Change in UV–Visible absorption spectra of methyl orange upon treatment with AgNPs, NaBH_4_, and AgNPs+NaBH_4_, **(B)** effect of different amount of nanoparticle on the degradation of dye, **(C)** effect of reaction time on the degradation of methyl orange using AgNPs at 2-min intervals, **(D)** change in absorption intensity of methyl orange at 464 nm at different time interval, upon addition of AgNPs catalyst.

Newly synthesized AgNPs were explored for their catalytic activity toward the degradation of methyl orange dye. Methyl orange is a polar dye highly soluble in water. It is used extensively as a dye in the fabric production as well as the pH indicator. The color of methyl orange is orange-red due to the presence of the azo group which absorbs visible radiations resulting in a clear peak at 464 nm in a UV-visible spectrum. The degradation of methyl orange can be examined spectrophotometrically. Methyl orange solution was mixed with solid AgNPs (2 mg/5 mL) and the resulted solution was stirred at 25°C for 15 min, and dye color change and absorption intensity were monitored. Degradation reaction proceeds immediately upon the addition of AgNPs that can be visualized by the fading orange color of the dye with a reduction in absorption intensity of the band at 464 nm as publicized in Figure 8A. The dark orange solution of methyl orange was being changed to a colorless solution within 15 min, and a significant decrease in the absorption intensity was also recorded. These results proposed that AgNPs cleaved the –N=N- functional group of methyl orange which subsequently decolorizes the target solution (Naseem et al., 2019; Raj et al., 2020). The ${\text{BH}}_{4}^{-1}$ and methyl orange adsorb on the surface of nanoparticles in aqueous solution. The hydrogen from ${\text{BH}}_{4}^{-1}$ acting as a source of hydrogen can absorb on the surface of AgNPs and attack on azo function group of methyl orange. The electron carrying NPS triggers the catalytic surface and activates azo bond of methyl orange which results as breakage of –N=N- bonds due to the acceptance of electron *via* catalyst, further the hydrogen released from ${\text{BH}}_{4}^{-1}$ and dyes get reduced on the surface of nanoparticles.

AgNPs with the properties of low volume to the high surface area can increase the rate of reduction for dye degradation. To optimize the amount of AgNPs catalyst, different amount of nanoparticles (1–3 mg) is treated with a constant concentration of methyl orange under the same conditions, an amendment in the absorption intensity was noted as depicted in Figure 8B. It showed the rate of decay of dye increase with the increase of the amount of the nano-catalyst (AgNPs) due to the high availability of the catalyst (high surface area) (Bogireddy et al., 2016). The reaction time has a foremost effect on the degradation process. The reaction was performed with a constant concentration of methyl orange (0.1 mM) at 25°C, and nano-catalyst (1 mg) reaction time is varied at 0–15 min. The change in the absorbance of methyl orange was recorded as shown in Figure 8C. The obtained results showed that the rate of methyl orange degradation is directly proportional to reaction time, and the equilibrium reaction rate is attained at optimal contact time. The change in the absorption intensity of methyl orange is plotted vs. reaction time (Figure 8D), which gives a straight line equation with *R*^2^ = 0.9927 and rate value of 0.0284 min^−1^. Table 3 lists various classic methods reported in the literature for the degradation of methyl orange concerning reaction time and catalyst dose. The results from the current study are also listed. Most of the reported methods are based on a complex chemical synthetic approach. In contrast, the current study is based on a simple, cheap, and more environment-friendly green synthesized AgNPs based nano-catalyst for efficient degradation of methyl orange.

**Table 3:** Comparison of the current method for degradation of methyl orange with reported methods.

**Entry**	**Catalyst**	**Catalyst dose (mg)**	**Time (min)**	**Degradation (%)**	**References**
1	MnFe_2_O_4_	2.5	90	100	Nguyen et al., 2011
2	Iron nanoclusters	1.25	360	95	Ebrahiminezhad et al., 2018
3	Co doped ZnO NPs	0.5	90	100	Ahmad, 2019
4	NiO NPs	5.0	05	95	Barzinjy et al., 2020
5	ZnO NPs	3.0	100	81	Gawade et al., 2017
6	AgNPs/Acacia nilotica	5.0	06	95	Shah et al., 2020
7	AgNPs/*Bistorta amplexicaulis*	2.0	15	98	Current work

## Conclusions

In this research work, we have developed highly stable green synthesized silver nanoparticles (AgNPs) by a simple, cheap, and ecological friendly biogenic route. The root extract of *Bistorta amplexicaulis* plant was acted as a reducing and capping agent for the stabilization of AgNPs. Aqueous extract of *Bistorta amplexicaulis* containing several biomolecules (such as carbohydrates, amino acid, phenol, and triterpenes) are effective for the stabilization of AgNPs. Newly synthesized AgNPs were characterized by several spectroscopic techniques. Hg^2+^ and Pb^2+^ ion produce a substantial effect on the absorption band of AgNPs accompanied by naked eye detectable color change. The change in color of AgNPs from dark brown to light yellow for Hg^2+^ and dark brown to light brown for Pb^2+^ due to complexation and aggregation of AgNPs in the solution. The limit of detection of Hg^2+^ and Pb^2+^ is in the micromolar range, comparing with the reported protocols. The designed sensing approach is highly selective for Hg^2+^ and Pb^2+^ as no interference is observed in the competitive experiments. Further, synthesized nanoparticles were admirably used for the recognition of the above-mentioned analytes in tap water samples without any pre-treatment process. Further AgNPs were found to be an effective catalyst for the degradation of methyl orange dye. This innovative protocol has numerous advantages over the published colorimetric sensors due to its simplicity, high selectivity, cost-effectiveness, and more importantly environmental friendliness. Thus, obtained sensing and catalytic potential of AgNPs suggest the use of *Bistorta amplexicaulis* stabilized nanoparticle for detection of heavy metal ions and degradation of methyl orange.

## Data Availability Statement

All datasets generated for this study are included in the article/Supplementary Material.

## Author Contributions

FA conceived the experiments and drafted the manuscript. FA and HK conducted all the experiments. HX helped to discuss the results. All authors contributed to the article and approved the submitted version.

## Conflict of Interest

The authors declare that the research was conducted in the absence of any commercial or financial relationships that could be construed as a potential conflict of interest.
