# Supplementary material for: Dual Colorimetric Sensor for Hg2+/Pb2+ and an Efficient Catalyst Based on Silver Nanoparticles Mediating by the Root Extract of Bistorta amplexicaulis
Source: Front Chem. 2020 Oct 22;8:591958. doi: 10.3389/fchem.2020.591958 (PMC7642621; doi:10.3389/fchem.2020.591958)
Supplement: Supplementary file 1 [file Data_Sheet_1.PDF]

Supplementary Material

1. Phytochemicals Tests

Preliminary qualitative phytochemical analysis was carried out to identify the secondary metabolites present in the aqueous extracts of root parts of *Bistorta amplexicaulis***.**

1.1 Test for carbohydrates

a. Fehling's test

About 2 mL of root extract of *Bistorta amplexicalius* plant was mixed with 2 mL of Fehling's A and B solution and then incubated in water bath for 20 min at 80 $^{\circ}$C.The presence of carbohydrate was indicated by brownish red color precipitate.

b. Benedict's test

About 1 mL of root extract of *Bistorta amplexicaulis* plant was treated with 1 mL Benedict's reagent and heat gradually. The presence of carbohydrates was indicated by orange red precipitate.

1.2 Test for amino acids

About 1 mL of root extract of *Bistorta amplexicaulis* plant was added in 1 mL of 0.25% Ninhydrin solution and then boiled for 5 minute. The presence of amino acids was confirmed by the formation of blue color.

1.3 Test for triterpenes

About 2 mL of root extract of *Bistorta amplexicaulis* plant was added in 2 mL chloroform for phase separation after adding concentrated sulphuric acid these solution allowed to stand for 2 minutes. The presence of triterpenes was confirmed by golden yellow color.

1.4 Test for phenols

About 2 mL of root extract of *Bistorta amplexicaulis* plant were mixed with 3-4 drops of ferric chloride solution. The formation of green color showed the absence of phenols.

1.5 Test for proteins

About 1 mL of root extract of *Bistorta amplexicaulis* plant was mixed with 5-6 drops of conc. nitric acid. The presence of proteins was confirmed by the formation of yellow color.

1.6 Test for flavonoids

About 2 mL of root extract of *Bistorta amplexicaulis* plant was mixed with 2 mL of 2.5 M sodium hydroxide. The absence of flavonoids was confirmed by blue color.

1.7 Test for alkaloids

Hager's test

About 1 mL of root extract of *Bistorta amplexicaulis* plant was mixed with saturated picric acid solution. The absence of alkaloids was confirmed by light brown color.

2.1 Salt stability

Stock solution of NaCl (1 M) was prepared in 25 mL distilled water. To evaluate the stability of NPs the different concentration ranging from 1×10^{-3} M to 1×10^{-2}M of this stock solution were tested and the UV-visible spectra was recorded.

2.2 Temperature stability

Bistorta amplexicalius AgNPs was subjected to heating at different temperature ranging from 20 ℃ to 100 ℃**.**

2.3 pH stability

The effect of pH on surface plasmon resonance peak of *Bistorta amplexicaulis* silver nanoparticles was evaluated by ranging pH from 2 to 10. For pH stability 1M sodium hydroxide and 1M sulphuric acid solution was used. For increasing the pH of the extract, sodium hydroxide solution was used and for decreasing pH sulphuric acid solution was added drop wise in the extract.

2.4 Time stability

Bistorta amplexicaulis stabilized AgNPs were kept at room temperature to evaluate the effect of time on stability of these nanoparticles and the UV-visible spectra were recorded from time of formation to 90 days.

2.5 Screening of metals

For detection of alkali (Na^{+1}, K^{+1}), alkaline earth (Mg^{2+}, Ca^{2+}, Ba^{2+}), transition-metal ions (Ni^{2+}, Cu^{2+}, Zn^{2+}, Hg^{2+}, Co^{2+}) and p-block metals (Al^{3+} and Sn^{2+}) *Bistorta amplexicaulis* AgNPs were used. About 1 mL of solution of various metals at 0.1 mM or 10^{-3} mol L^{-1} were added into 1 mL of two time diluted solution of *Bistorta amplexicaulis* AgNPs. These mixtures were stored at room temperature for few minutes under observation and then UV–visible absorption spectra were recorded. The picture was taken by digital camera.

2.6 Concentration study

To estimate the sensitivity of *Bistorta amplexicaulis* AgNPs toward Hg^{2+} and Pb^{2+}solution of various concentrations were prepared from stock solution. After preparing these solutions 1 mL of every solution was added in 1 mL of *Bistorta amplexicaulis* AgNPs. The reaction mixture was stored at room temperature and recorded UV–visible absorption spectra.

2.7 Competitive experiments

To estimate the detection of selected metals in the presence of other metals interference study was carried out. For this purpose 1 mL selected metals Hg^{2+} (80 μM) and Pb^{2+} (100 μM) along with each metal ion including, Cu^{2+}, Zn^{2+}, Mg^{+2}, $Ni^{2+,}$ $Na^{1+,}$ $K^{1+,}$ Al^{3+}, Ba^{2+}, Ca^{2+} and Sn^{2+} into a 1 mL *Bistorta amplexicaulis* AgNPs solution. Thereafter, the UV-VIS spectra were recorded.

2.8 Effect of pH

This study was performed to explore the effect of pH on the sensitivity of Hg^{2+} and Pb^{2+} in biosynthesized *Bistorta amplexicaulis* AgNPs sensing system. This step was carried out by addition of HCl and KOH in reaction mixture. Different pH (ranging from 2.0 to 12.0) maintained and noted the UV Spectra.

2.9 Job plot

To determine binding stoichiometry solution of different concentration of NPs with Hg^{2+} and Pb^{2+} (ranging from 0.1-0.9) were used.

Results and Discussion

3.1 Preliminary Screening of Phytochemicals

The phytoconstituent screening *Bistorta amplexicalius* root extract was performed by conventional phytochemical analysis and obtained results were summarized in table 1. The presents study revealed that aqueous extract of *Bistorta amplexicalius* contain various secondary metabolites such as carbohydrates, amino acid, triterpenes phenols etc. Test for alkaloids, steroids and flavonoids give negative results which demonstrates that's these classes of secondary metabolites are not present in the aqueous extract of *Bistorta amplexicalius.*

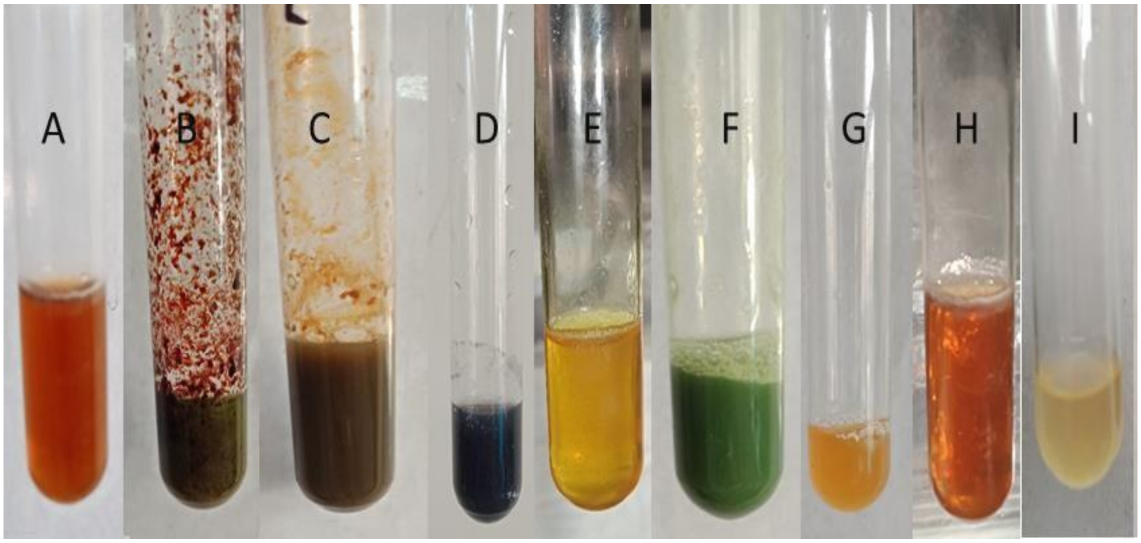

Figure S1: Photograph of phytochemical screening (A) *Bistorta amplexicalius* root extract, test for carbohydrates (B, C), amino acid (D), triterpenes (E), phenols (F), protein (G), flavonoids (H), alkaloids (I).

Table 1: Phytoconstituent screening of aqueous extract of *Bistorta amplexicalius* root extract.

Sr. No	Phytoconstituents	Result
1.	Carbohydrate	Positive
2.	Amino acid	Positive
3.	Proteins	Positive
4.	Triterpenes	Positive
5.	Phenols	Positive
6.	Alkaloids	Negative
7.	Flavonoids	Negative

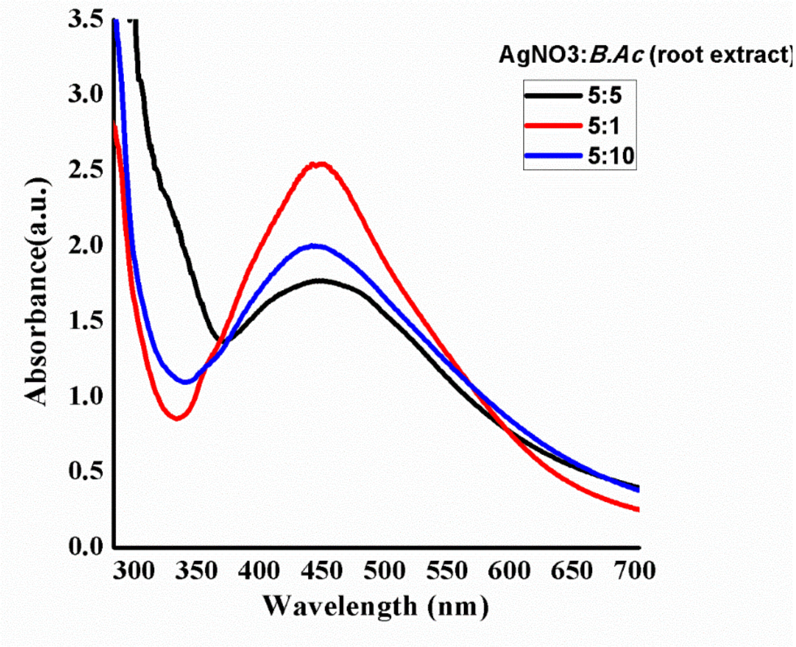

Figure S2: Optimization of ratio for the synthesis of AgNPs

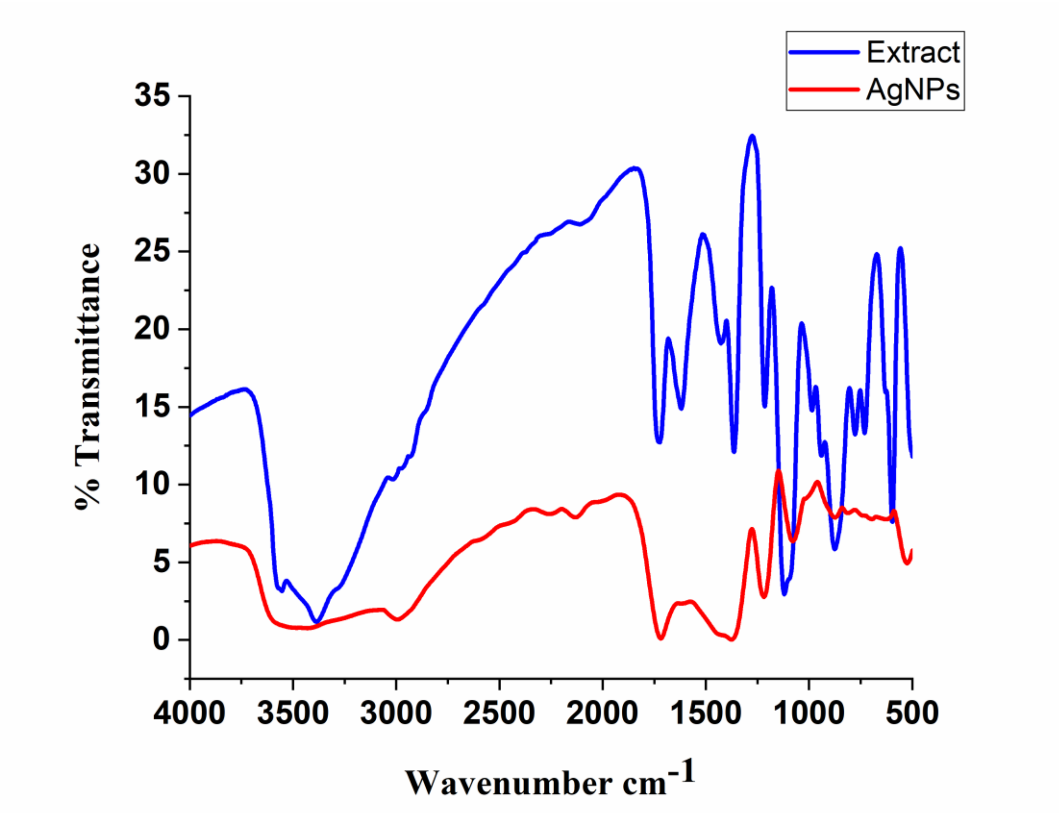

Figure S3: FTIR spectrum of plant extracts and synthesized nanoparticles

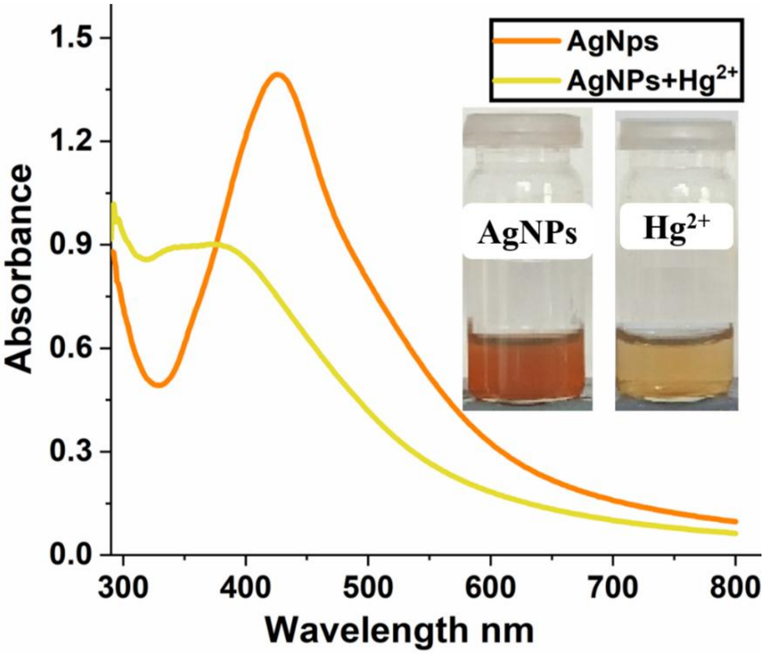

Figure S4: Change in absorption intensity of green synthesized nanoparticles upon interaction with Hg^{2+}

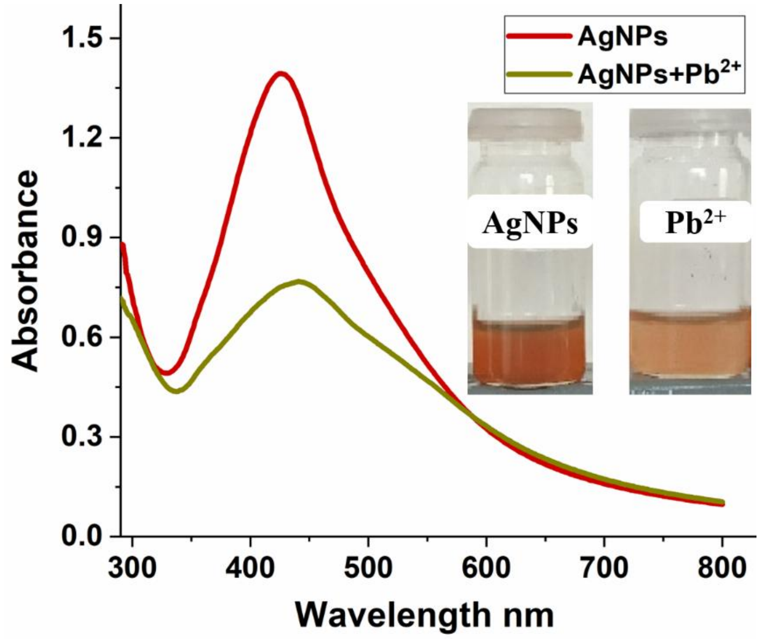

Figure S5: Change in color and absorption intensity of AgNPs upon addition of Pb^{2+}

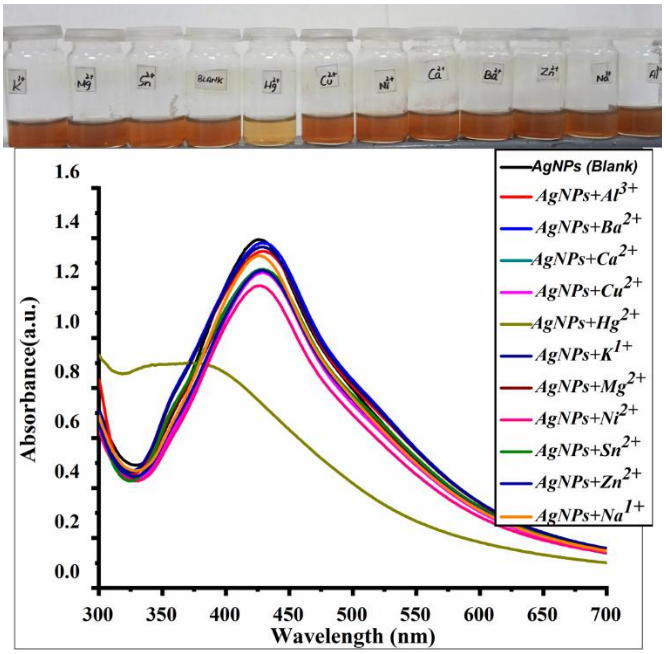

Fig S6: Change in absorption and color of AgNPs upon interaction with Hg^{2+}

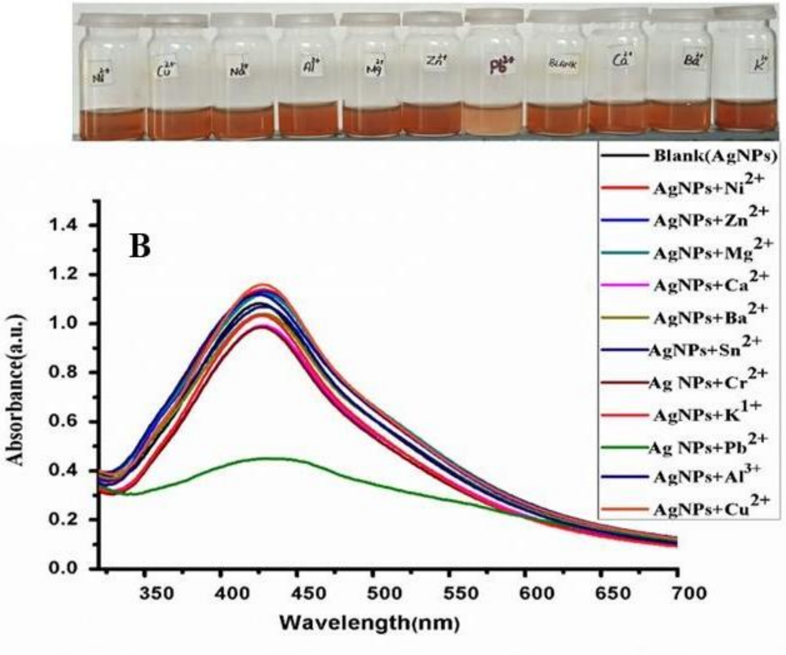

Figure S7: Colorimetric recognition of Pb^{2+} ion using AgNPs

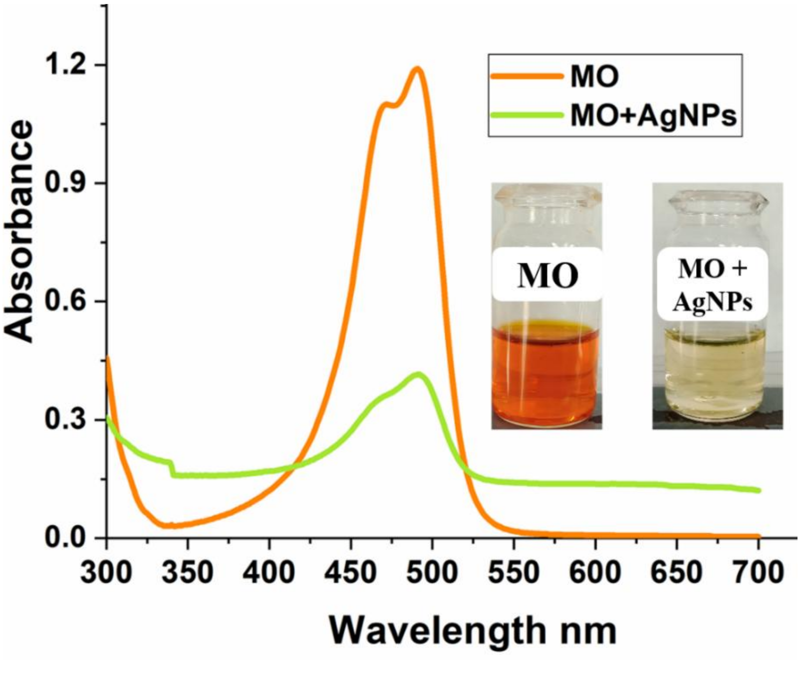

Figure S8: Degradation of methyl orange using AgNPs: colorimetric and absorption change
