# Supplementary material for: Dual Colorimetric Sensor for Hg2+/Pb2+ and an Efficient Catalyst Based on Silver Nanoparticles Mediating by the Root Extract of Bistorta amplexicaulis
Source: Front Chem. 2020 Oct 22;8:591958. doi: 10.3389/fchem.2020.591958 (PMC7642621; doi:10.3389/fchem.2020.591958)
Supplement: Supplementary file 2 [file Data_Sheet_2.PDF]

Graphical Abstract

Dual Colorimetric Sensor for Hg^{2+}/Pb^{2+} and an Efficient Catalyst Based on Silver Nanoparticles Mediating by the Root Extract of *Bistorta Amplexicaulis*

Farid Ahmed[1, 2], **Humaira Kabir**[3], **Hai Xiong**[1*]

[1] Institute for Advanced Study, Shenzhen University, Shenzhen, 518060, P. R. China.
[2] College of Physics and Optoelectronic Engineering, Shenzhen University, Shenzhen, 518060, P. R. China.
[3] Department of Chemistry, Women University of Azad Jammu and Kashmir, Bagh, Pakistan.

*** Correspondence:** hai.xiong@szu.edu.cn

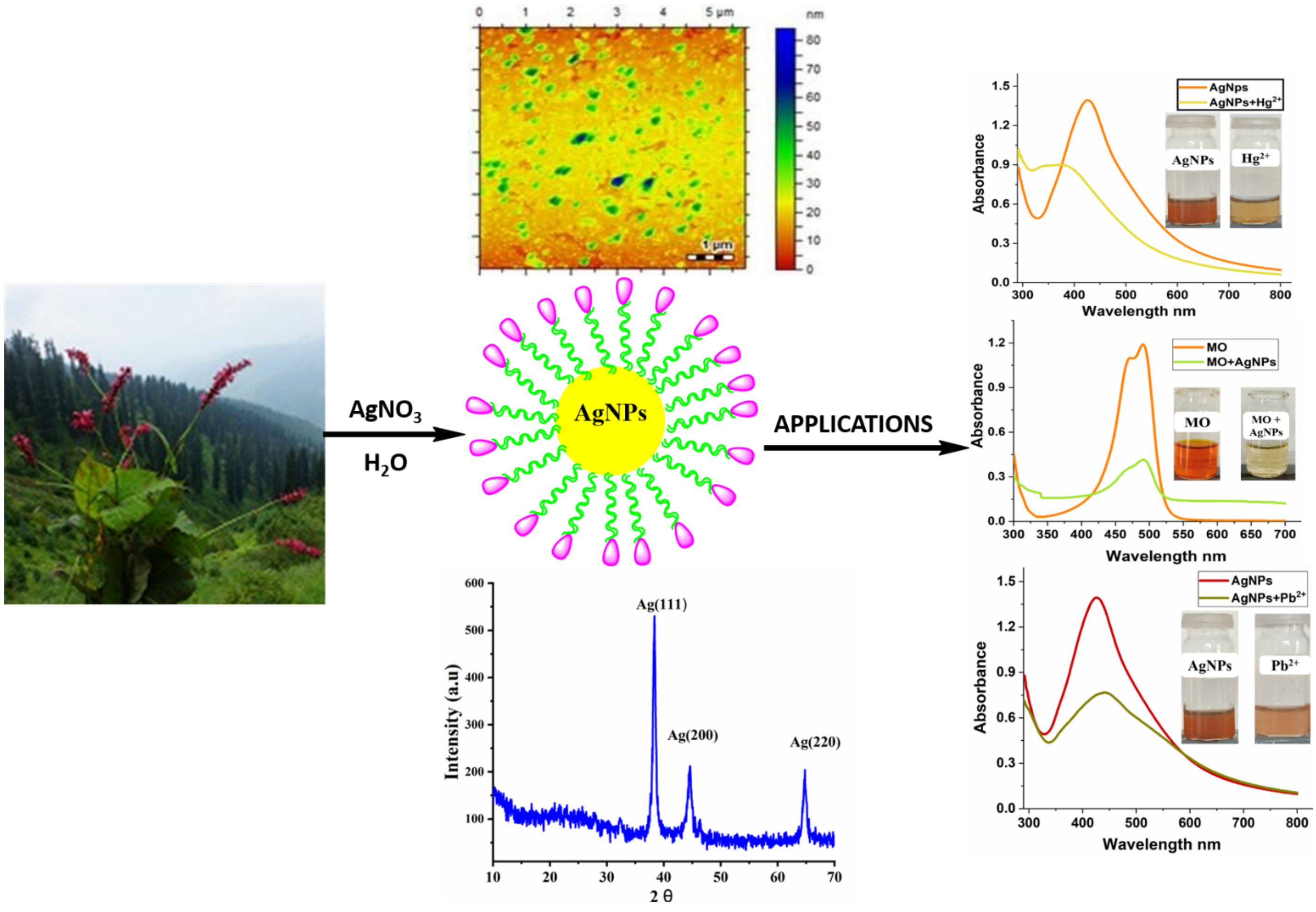

Green synthesized AgNPs were found to be excellent dual colorimetric sensor for Hg^{2+} and Pb^{2+} with a limit of detection of 0.8µM and 0.2µM respectively. AgNPs as colorimetric sensor offer qualitative and quantitative information by naked-eye visibility without using expensive equipment.
