# Supplementary material for: Dual Colorimetric Sensor for Hg2+/Pb2+ and an Efficient Catalyst Based on Silver Nanoparticles Mediating by the Root Extract of Bistorta amplexicaulis
Source: Front Chem. 2020 Oct 22;8:591958. doi: 10.3389/fchem.2020.591958 (PMC7642621; doi:10.3389/fchem.2020.591958)
Supplement: Supplementary file 3 [file Data_Sheet_3.PDF]

Research Highlights

- A highly selective and an inexpensive colorimetric sensor for the detection of Hg^{2+}(1×10^{-6}–1×10^{-7}M) and Pb^{2+}(1×10^{-6}–1×10^{-8}M) with good linearity was developed by using AgNPs synthesized from *Bistorta amplexicalius*(root extract).
- AgNPs as colorimetric sensor offer qualitative and quantitative information by naked-eye visibility without using expensive equipment.
- Utilization of plant extract due to its simplicity, non-toxicity, easy availability, relative reproducibility, eco-friendly, low cost and higher effectiveness. It also does not require intense maintenance of laboratory cultures for nanoparticles synthesis.
- Silver nanoparticles mediating by *Bistorta amplexicalius* were found to be highly effective catalyst for the degradation of methyl orange dye.
