# Supplementary material for: Dual Colorimetric Sensor for Hg2+/Pb2+ and an Efficient Catalyst Based on Silver Nanoparticles Mediating by the Root Extract of Bistorta amplexicaulis
Source: Front Chem. 2020 Oct 22;8:591958. doi: 10.3389/fchem.2020.591958 (PMC7642621; doi:10.3389/fchem.2020.591958)
Supplement: Supplementary file 4 [file Data_Sheet_4.PDF]

Aug 6th, 2020

Dear Editors, *Froniters in Chemistry*

Prof. Steve Suib

Subject: **Dual Colorimetric Sensor for Hg^{2+}/Pb^{2+} and an Efficient Catalyst Based on Silver Nanoparticles Mediating by the Root Extract of *Bistorta Amplexicaulis***

In our manuscript, silver nanoparticles(AgNPs) were synthesized firstly by using root extract of *Bistorta amplexicaulis.* Green synthesized AgNPs as dual colorimetric sensor offer qualitative and quantitative information by naked-eye visibility without using expensive equipment. We think that this work describes the fabrication technology of green synthesized nanomaterials and its application. Green synthesized AgNPs exhibit high selectivity and potential applications to be molecular luminescence / sensors or effective catalyst.

Therefore, we wish to submit our manuscript as an article in *Froniters in Chemistry*. Your kind consideration is highly appreciated.

Research Highlights

- A highly selective and an inexpensive colorimetric sensor for the detection of Hg^{2+}(1×10^{-6}–1×10^{-7}M) and Pb^{2+}(1×10^{-6}–1×10^{-8}M) with good linearity was developed by using AgNPs synthesized from *Bistorta amplexicalius*(root extract).
- AgNPs as colorimetric sensor offer qualitative and quantitative information by naked-eye visibility without using expensive equipment.
- Utilization of plant extract due to its simplicity, non-toxicity, easy availability, relative reproducibility, eco-friendly, low cost and higher effectiveness. It also does not require intense maintenance of laboratory cultures for nanoparticles synthesis.
- Silver nanoparticles mediating by *Bistorta amplexicalius* were found to be highly effective catalyst for the degradation of methyl orange dye.

We respectfully suggest the following expert reviewers for our manuscript:

- Hangxiang Wang, School of Medicine, Zhejiang University, expert in “Supramolecular nanoassembly, Prodrug”, wanghx@zju.edu.cn
- Yiwen Li, College of Polymer Science and Engineering, Sichuan University, expert in “Click chemistry, bio-inspired polymeric materials”, ywli@scu.edu.cn
- Muhammad Ateeq, Abdul Wali Khan University, expert in “Nanoparticle based sensor”, ateeq672@yahoo.com
- Yanlong Gu, School of Chemistry and Chemical Engineering, Huazhong University of Science and

Technology, expert in “Green synthesis and environmental friendly catalysis”, klgyl@hust.edu.cn

- Jianxun Ding, Key Laboratory of Polymer Ecomaterials, Changchun Institute of Applied Chemistry, Chinese Academy of Sciences, expert in “Nanomedicine, shell-stacked nanoparticle (SNP)”, jxding@ciac.ac.cn

Prof. Hai Xiong
Institute for Advanced Study,
Shenzhen University, Nanhai Avenue 3688, Shenzhen, China 518060
Telephone: (0086)795-26948485, E-mail: hai.xiong@szu.edu.cn
